# Cellular senescence or stemness: hypoxia flips the coin

**DOI:** 10.1186/s13046-021-02035-0

**Published:** 2021-07-29

**Authors:** Daniel Otero-Albiol, Amancio Carnero

**Affiliations:** 1 Instituto de Biomedicina de Sevilla, IBIS, Hospital Universitario Virgen del Rocío, Universidad de Sevilla, Consejo Superior de Investigaciones Científicas, Avda. Manuel Siurot s/n, 41013 Seville, Spain; 2 CIBER de CANCER, Instituto de Salud Carlos III (ISCIII), Madrid, Spain

**Keywords:** hypoxia, oxygen, cellular senescence, immortalization, cancer, stemness, dedifferentiation

## Abstract

Cellular senescence is a complex physiological state whose main feature is proliferative arrest. Cellular senescence can be considered the reverse of cell immortalization and continuous tumor growth. However, cellular senescence has many physiological functions beyond being a putative tumor suppressive trait. It remains unknown whether low levels of oxygen or hypoxia, which is a feature of every tissue in the organism, modulate cellular senescence, altering its capacity to suppress the limitation of proliferation. It has been observed that the lifespan of mammalian primary cells is increased under low oxygen conditions. Additionally, hypoxia promotes self-renewal and pluripotency maintenance in adult and embryonic stem cells (ESCs), induced pluripotent stem cells (iPSCs) and cancer stem cells (CSCs). In this study, we discuss the role of hypoxia facilitating senescence bypass during malignant transformation and acquisition of stemness properties, which all contribute to tumor development and cancer disease aggressiveness.

## Background

Cellular senescence is a complex physiological state of the cell, whose main features are proliferative arrest, changes in chromatin structure, specific epigenetic modifications, metabolic changes, high acid β-galactosidase activity, an increased secretion phenotype and organelle alterations. Senescence can be induced by intrinsic and extrinsic stimuli such as telomere shortening and DNA damage response, oncogenic signaling detection, oxidative stress, or anticancer therapies [1, 2]. Cellular senescence is necessary for the regulation of different physiological processes, such as tumor suppression, development of embryo structures, wound repair and cellular reprogramming in tissue repair [3–5]. Furthermore, cellular senescence correlates with tissue aging and is involved in the deleterious effects of the diseases associated with it [6].

Despite the physiological relevance of senescence, it is still unknown whether hypoxia, which is a feature of every tissue in the organism, modulates its initiation regulating its full normal development. Thus, it is unclear whether hypoxia works as a stress-inducing physiologically regulated senescence or is able to promote proliferation and immortalization prior to malignant transformation. It has been shown that hypoxia contributes to tumor malignancy [7]. Moreover, hypoxia supports pluripotency maintenance and self-renewal in embryonic stem cells (ESCs), adult stem cells, induced pluripotent stem cells (iPSCs) and cancer stem cells (CSCs) [8–10]. In this review, we bring together senescence and hypoxia and study their relationship with immortalization, acquisition of stemness and malignant properties.

## Main text

### Main features of cellular senescence

#### Cell cycle arrest

The cell cycle is a process divided into four phases (G1, G2, S and M). Progression from one phase to the next depends mainly on the action of cyclins and cyclin-dependent kinases (CDKs). Ten different cyclins that form four different groups (A, B, D and E) are specifically synthesized and degraded during each phase of the cell cycle, regulating the activity of CDKs. In general, senescent cells present G1-DNA (2N) because of the expression of CDK inhibitors (CKIs) belonging to the INK4 and CIP/KIP families. These CKIs inhibit CDK2, CDK4 and CDK6, which control the progression from G1 to S, and CDK1 in G2/M. Furthermore, during G1 phase of the cycle, there is a checkpoint, known as the restriction point or R point that reunites all of the intrinsic and extrinsic signals to allow or prevent transition to S phase. When cellular senescence is induced, several factors regulate retinoblastoma protein (pRB) to block the G1/S transition. Among these factors, the high expression of CKIs is very important [11, 12].

Classically, cell cycle stop associated with cellular senescence has been considered irreversible. However, some authors support the possibility of return to proliferation under specific genetic or transcriptomic conditions [13]. During replicative senescence, inactivation of p53 allows proliferation if *p16*^*INK4a*^ expression is low [14]. The punctual loss of pRB proteins allows cell cycle progression in *RAS*-induced senescence. It has also been shown that the H3K9 demethylating proteins JMJD2C and LSD1 are able to promote proliferation after oncogene-induced senescence (OIS) [15].

#### Apoptosis resistance

Senescent cells are also characterized by survival signaling, which promotes resistance to apoptosis. Under normal conditions, severe DNA damage and other kinds of stress can promote the proapoptotic activity of p53 and the expression of its transcriptional targets, *NOXA* and *BAX* [16]. However, senescent cells show high activity of antiapoptotic molecules such as BCL-2, BCL-xL, BCL-w, PI3K and p21^CIP1^ [1, 2]. *p21*^*Cip1*^ is essential for survival signaling, as it has been shown that *p21*^*Cip1*^-deficient mice accumulate DNA damage and induce apoptosis [17]. Additionally, *in vivo* inhibition of BCL-W and BCL-XL can eliminate senescent cells in the lung and epidermis [18].

#### Chromatin changes

Accumulated DNA damage in senescent cells has consequences on chromatin structure. Thus, nuclear foci denominating DNA segments with chromatin alterations reinforcing senescence (DNA-SCARS) can be permanently found in senescent cells. Although these DNA-SCARSs contain active DNA repair proteins, such as CHK2 or p53, as well as other transitory DNA repair foci, there are no single strand DNAs, DNAs in synthesis or PML nuclear bodies, and they lack RAP and RAD5 proteins. These foci can promote the senescence-associated secretory phenotype (SASP) and stop the cell cycle. To do this, the foci need p53 and pRB [19]. In senescent cells, it is also possible to observe senescence-associated heterochromatin foci (SAHFs). These structures are located in the promoters of cell cycle genes regulated by E2F, silencing them in collaboration with pRB [20, 21]. SAHFs are generated by reorganization of epigenetic repressor modifications such as H3K9, HP1 or macroH2A without an H1 linker [22, 23]. Another chromatin alteration observed in senescence is satellite distensions associated with senescence (SADSs), which consist of decondensation of the constitutive heterochromatin formed by the pericentromeric satellite DNA. This alteration appears prior to SAHFs and differs from them in that it conserves epigenetic canonical marks and is independent of senescence-specific signaling pathways [24].

#### Epigenetic modifications

Cells suffer great changes during acquisition of a senescent phenotype. These alterations result in changes in accessibility to chromatin and, accordingly, affect gene expression. DNA methylation and histone modifications are altered during cellular senescence [25].

Replicative senescence is associated with globally hypomethylated DNA in CpG sites, except for specific sites that are hypermethylated. This hypomethylation is associated with deregulation of the DNMT1 enzyme acquired during successive DNA replications and is associated with an increase in p16^INK4a^ and p21^CIP1^. However, in premature senescence, such as that induced by doxorubicin treatment, radiation or oncogenic RAS overexpression, DNA hypomethylation is not observed [25–28].

Histones suffer posttranslational modifications in their amino-terminal tail, which changes their interaction with nucleosomes and participates in the regulation of chromatin structure [29]. Replicative senescence is associated with a decrease in global histones and, consequently, reduced modified histones. Thus, it has been suggested that the repressor Rap1 leaves its binding sites at chromosome ends to bind histone coding genes. Additionally, a general decrease in the histone modifications H4K16ac, H3K4me3, H3K9me3 and H3k27me3 and a general increase in H3K9ac and H4K20me3 along with p-H2AX have been reported. Specifically, p-H2AX is associated with telomere shortening and colocalizes with double string break (DBS) repair machinery [25].

Additionally, it has been shown that macroH2A1 increases PARP1 activity, promoting SASP via the PARP/NFkB pathway. In this case, a positive feedback loop is generated because SASP factors promote macroH2A1 expression [30, 31]. At this respect, macroH2A1 presence can be determinant to senescence in tumors as it plays an important role in regulation of SASP and it modulates CSC identity and chemoresistance as it has been described, especially, in hepatocellular carcinoma [32–35]. However, this role of macroH2A1 is not exclusive to CSCs, as it is implied in stem cell fate and reprogramming [36–41] among others. MacroH2A1 is one of the chromatin regulators linked to many of the processes linked such as stemness, senescence, SAPS, hypoxia or DNA damage, being involved in all of them. However, the specificity of the signal regulating the specific mechanism altering each physiological process is still poorly known.

Histone modifications can recruit chromatin remodeling enzymes, which use ATP hydrolysis to change nucleosome positions [29]. This process has also been associated with cellular senescence. For example, ARID1B, which is part of the remodeling complex SWI/SNF, can produce DNA damage and induce reactive oxygen species (ROS) production. In this way, *p53*, *p21*^*CIP1*^ and *p16*^*INK4a*^ expression is increased, and senescence is initiated [42, 43]. Furthermore, the SWI/SNF complex facilitates Polycomb repressive complex 2 (PCR2) evacuation, which can activate the expression of genes encoded by the *INK4a/ARF* locus [44].

#### Metabolic changes

Despite their nonproliferative state, senescent cells present high metabolic activity. This is due to a high demand for energy and cellular components to satisfy the senescent phenotype [1, 45, 46]. Catabolism in senescent cells focuses on high consumption of glucose to generate ATP. Glycolysis increases with oncogenic or genotoxic stimuli and during replicative senescence. Furthermore, the presence of dysfunctional mitochondria, which is a common feature of cellular senescence, affects the production of ATP and the NAD^+^/NADH ratio, which drives proliferative arrest. Additionally, AMPK and NFkB signaling are commonly altered catabolic pathways in senescent cells [45, 47, 48].

On the other hand, during cell senescence, anabolism is focused on the synthesis of proteins, contributing to SASP, and lipids to form new membranous organelles and stimulate autophagy. Glycogen synthesis is also increased in human senescent fibroblasts and during the aging process in different tissues. Additionally, replicative stress is notable in the DNA synthesis process. Related to these anabolic processes, the signaling pathways most altered during cellular senescence are GSK3, ATM, SREBP1, and mTOR [45, 46, 49].

#### Senescence associated secretory phenotype (SASP)

The senescent-messaging secretome is formed by the pool of cytokines, chemokines and proteases synthesized and secreted by senescent cells. These molecules help cells communicate with surrounding tissues. SASP is a heterogenic and pleiotropic phenotype that depends on genetic context, cell type and microenvironment. Therefore, its functions are also diverse and include positive and negative physiological aspects [1, 50].

The main functions of SASP are autocrine and paracrine reinforcement of the cell cycle stop and an increase in the senescent phenotype. In this process, molecules such as the interleukins IL6 and IL8 or receptors such as IL6R and CXCR2 form a positive feedback loop to promote cellular senescence. This signaling also supposes a tumor suppressor mechanism that stops the growth of nontransformed cells [51–53]. SASP is relevant in tissue remodeling, senescence during embryo development, tissue repair and cellular plasticity [5]. Furthermore, senescent cells can recruit the immune system, contributing to the elimination of premalignant and senescent cells. This is possible because of the secretion of proinflammatory interleukins [54].

SASP also contributes to tumor development through the secretion of factors such as VEGF, which promotes angiogenesis or recruits immature myeloid cells. This produces an immunosuppressant effect that is relevant in the development of some tumors, such as prostate or liver [55–57]. On the other hand, the proinflammatory activity of the SASP is related to deleterious effects of aging due to the accumulation of senescent cells in tissues. This concept is called “inflammaging” [58].

The functions, composition and regulation of the SASP are also diverse and context-dependent. Nevertheless, there are some key factors that are relevant in this phenotype, such as NFkB, C/EBPB, mTOR, MLLT1, GATA4, p38 MAPK and some effectors of the DNA damage response (DDR). NFkB and C/EBPB constitute a positive feedback loop with IL6 and IL8, which reinforces the secretory phenotype activating NFkB and C/EBPB again [51, 53]. Multiple pathways regulating SASP converge in mTOR, which can promote the action of IL1a, a key factor in the development of SASP. Furthermore, the diversity of secreted molecules can be classified into two secretomes: the inflammatory secretome, which is mainly regulated by IL1, and the TGFB secretome, which depends on yuxtacrine signaling of NOTCH. Although most of the molecules participating in SASP are soluble proteins secreted into the extracellular medium, there are also transmembrane proteins that are cleaved and factors secreted in exosome-like vesicles [1, 50, 58].

### Structural and morphological alterations in cellular senescence

#### Changes in autophagy and lysosomes

Autophagy is a cellular process that enables the recycling of cytoplasmic components, including those in vacuoles, and their degradation in lysosomes. Senescent cells present high autophagic activity due to the high energetic demand and abundance of aberrant cellular components. Furthermore, it is thought that autophagy activation can initiate cellular senescence [1, 20]. As an example, inactivation of mTORC1 during nutrient starvation initiates autophagy and the recycling of cytoplasmic material [59]. This is because mTOR can form a compartment called the TOR autophagy spatial coupling compartment (TASCC), which appears during RAS-induced senescence. Under these conditions, mTOR inhibition decreases SASP proteins, IL6 and IL8 [60].

Even though cells need the activation of autophagy for the proper development of SASP, the synthesis and degradation of proteins are unbalanced in senescent cells, as is the case for other cellular components. This, along with other kinds of stress that are frequent in senescence (oxidative stress, accumulated mutations, lack of chaperons), drives the generation of toxic aggregates made of covalently bound lipids and proteins, increasing the senescent phenotype [1, 45].

To maintain homeostasis, autophagy might be accompanied by high lysosomal activity. Therefore, lysosomal content and activation of lysosomal proteins are increased in senescent cells. It is under discussion whether this is due to biogenesis or the accumulation of these organelles. Inside the lysosomes of senescent cells, there is high activity of the enzyme β-galactosidase. This activity is extensively used as a senescence biomarker and is measured using an artificial reactant known as X-Gal (5-bromo-4-chloro-3-indol-β-D-galactopyranoside) dissolved at an acid pH. The β-galactosidase enzyme is translated from one of the transcripts of the GLB1 gene. Although there are some mechanisms proposed for the transcriptional regulation of this gene and its posttranslational modifications, why it is overexpressed in senescent cells has not been determined [2, 20].

Some types of cells, such as tissue mature macrophages or osteoclasts, present high acid β-galactosidase activity, which is the most commonly used senescent marker along with *p16*^*INK4a*^ overexpression [61, 62]. Furthermore, there is solid evidence that supports acid β-galactosidase activity as a physiological phenomenon. Skin aging has been associated with the accumulation of senescent cells in this tissue, which shows high acid β-galactosidase activity [63]. Acid β-galactosidase activity has also been observed in OIS in premalignant lesions in *in vivo* models and patient samples [3]. Additionally, acid β-galactosidase activity is observed in other physiological processes, such as tissue repair during embryonic development [64, 65].

#### Dysfunctional mitochondria

Dysfunctional mitochondria accumulate in senescent cells, mainly because of aberrant mitophagic activity. Mitochondria in senescent cells suffer morphological and functional changes, and they change fusion-fission dynamics in favor of the fusion process. Additionally, mitochondria in senescent cells change their membrane potential, which is responsible for proton leakage and an increase in ROS, a factor that is crucial in cellular senescence [1, 2, 66].

#### Senescence-associated morphology

Senescent cells present a flat and enlarged morphology *in vitro*. This morphology can be a consequence of the overexpression of *CAV1* under stress conditions. This gene encodes CAVEOLIN1, a component of the cholesterol-rich microdomains of the plasma membrane. In human epithelial cells under oxidative stress, p38 MAPK promotes transcriptional activation of *CAV1,* producing morphological changes in the cells [67]. At the morphological level, it is also possible to observe multinucleated senescent cells. These cells have a defective nuclear lamina because LaminB1 is lost when p53 or pRB is activated [68]. Furthermore, it has been demonstrated that LaminB1 can be used as a biomarker for the detection of damaged cells and during skin regeneration in humans [69].

### Mechanisms that trigger cellular senescence

#### Replicative senescence and DNA damage

Replicative senescence was first described decades ago, when Dr. Leonard Hayflick observed that the number of duplications of human diploid fibroblasts was limited [70]. Later, replicative senescence was observed in multiple cell types, including endothelial cells, keratinocytes, lymphocytes, adrenocortical cells, chondrocytes and smooth muscle vascular cells [71].

Replicative senescence is caused by telomere shortening. Telomeres are structures formed by repetitions of the nucleotide sequence ‘TTAGGG’ and the proteins associated with them at the end of the chromosomes [72]. These structures protect chromosomes from damage and fusion with adjacent chromosomes [73]. Furthermore, telomeres shorten in every cellular duplication due to the inability of DNA polymerases to replicate the end of DNA strings [74]. This was demonstrated in 1990 in human fibroblasts, and later, it was detected *in vivo* in lymphocytes and cells from liver, skin, blood, and colon tissues [71, 75]. This inability to replicate chromosome ends is solved by the action of the enzyme telomerase (TERT). TERT is an inverse transcriptase that can add ‘TTAGGG’ repetitions to the end of chromosomes using an RNA molecule as a template [76]. The ectopic expression of *TERT* bypasses senescence in human fibroblasts [77]. However, in *Tert-*deficient mouse embryonic fibroblasts (MEFs), replicative senescence is not induced earlier. After several generations, the offspring of these *Tert*-deficient mice present critical short telomeres, which produces genomic instability and a premature aging phenotype, including short lifespan, difficulties in responding to stress and an increase in the appearance of malignant lesions [78, 79]. However, reactivation of *Tert* mitigates this phenotype [80].

Cells expressing telomerase enzymes differ among mammalian species. In humans, *TERT* is expressed in embryonic stem cells (ESCs), some adult stem cells and a few somatic cells. In mice, by contrast, it is possible to find Tert activity in several types of adult somatic cells, including fibroblasts. Despite this and the fact that telomeres of mice are 5-10 times longer, their lifespan is 30 times shorter [81–83]. Thus, in comparative studies in mammals, the length of telomeres negatively correlates with body size and, in contrast, positively correlates with telomerase activity. This could suggest an evolutionary tendency to short telomeres and low telomerase activity in favor of tumoral suppression. In humans, telomere shortening has been associated with stress- and aging-related pathologies, but not in a causal way. For example, some genetic disorders, such as congenic dyskeratosis, produce dysfunctional telomeres and aging-associated symptoms, such as premature lung fibrosis and cirrhosis [71, 84].

Cells can detect telomere shortening and start the DNA damage response (DDR), which leads to the cell cycle stopping to repair the damage. If this damage is irreparable, signaling drives cells into replicative senescence [73]. The DDR can be activated in cells when other DNA perturbations appear, and they are sensed to severe DNA damage, such as double string breaks (DSBs). Some of these perturbations are common under conditions of oxidative stress, oncogenic stress, ionizing radiation and chemotherapy. Then, the cellular response starts with ATM and ATR kinases phosphorylating H2AX histones around DNA damage and recruiting the DNA repairing complex. Another targets of ATM kinase are CHK1, CHK2, the adaptor protein 53BP1 and the regulator of checkpoint MDC1 [2, 6, 85].

#### Effectors of proliferative arrest on cellular senescence

p53. Different stimuli can lead to the phosphorylation of different residues of the p53 protein and its stabilization. This induces the cell cycle to stop through activation of CKI p21^CIP1^ and interaction with the p16^INK4a^/pRB pathway. p21^CIP1^ can block the activity of CDK2, CDK4 and CDK6, resulting in hypophosphorylated levels of pRB and proliferative arrest [1, 86]. Moreover, p53 protein is regulated by ARF. ARF helps to maintain p53 levels by binding to HDM2 and blocking its interaction with p53 and its degradation [87]. Additionally, p53 can initiate DNA repair and promote apoptosis, cellular senescence and metabolic changes under a wide range of endogenous and exogenous stimuli [88]. p53 activity is essential for replicative senescence in human and mouse fibroblasts. This is not the case for p21^CIP1^, which is necessary for cellular senescence in humans but not in mice [89]. However, *p21*^*CIP1*^-deficient mice are more predisposed to develop tumors [90]. Moreover, the relevance of p53 as a tumor suppressor is confirmed by its frequency of mutations in most types of human tumors [88].

##### CDK inhibitors

As mentioned before, CKIs belong to two different families. On the one hand, the INK4 family is composed of p16^INK4a^ (*CDKN2A*), p15^INK4b^ (*CDKN2B*), p18^INK4^c (*CDKN2C*) and p19^INK4d^ (*CDKN2D*), which bind specifically to CDK4 and CDK6, producing a halosteric change in them and blocking the binding of type D cyclins. On the other hand, the CIP/KIP family is composed of p21^CIP1^ (*CDKN1A*), p27^KIP1^ (*CDKN1B*) and p57^KIP2^ (*CDKN1C*), which can bind to CDK-cyclin complexes and inhibit them. All of these proteins are important tumor suppressors because of their role in proliferative arrest [91, 92].

Among this group of CKIs, p16^INK4a^ is especially important because of its relationship with pRB. The p16INK4a/pRB pathway responds directly to several stimuli and indirectly to DNA damage and p53 signaling, which can stop the cell cycle [21]. Specifically, pRB works as a tumor suppressor because it joins and inhibits E2F factors, which regulate the transcription of genes necessary for DNA replication and the cell cycle [93].

The levels of p16INK4a expression increase considerably with the number of replications of cells *in vitro*; its deletion is usual in immortalized cells, and the loss of its function, by punctual mutation or deletion, is one of the most common mutations in human tumors [20, 94]. Furthermore, *p16*^*INK4a*^ silencing through methylation of its promoter has been observed in multiple types of human tumors [95].

*p16*^*INK4a*^*, p15*^*INK4b*^ and *ARF* share the same locus, called *INK4a/ARF*. Although p15^INK4b^ is similar, functionally and structurally, to p16^INK4a^, the first has its own open reading frame independent of the other two genes. Even though *p16*^*INK4a*^ and *ARF* share 2 of their 3 exons, they do not present any homology in their amino acid sequences because these proteins are coded in alternative reading frames. Their transcription starts in different locations, and they have different first exons. ARF is also a relevant tumor suppressor gene, as it helps to maintain p53 protein levels [92].

Thus, regulation of the *INK4a/ARF* locus is crucial for cell cycle stopping and cellular senescence. In normal proliferative cells, the *INK4a/ARF* locus is silenced due to the polycomb group of proteins (PcG) [96]. Alteration of PcG or the loss of function of some of its components, such as CBX7, BMI1 or EZH2, leads to a loss of silencing epigenetic modifications in the locus and, consequently, to the activation of *p16*^*INK4a*^ and *ARF* expression and cellular senescence. Other epigenetic modifiers that act over the locus *INK4a/ARF* are CTCF, p300, MLL1 and ZRF1 [1, 20, 96]. The *INK4a/ARF* locus is regulated by other alternative mechanisms, among which transcription factors are also relevant. Some transcriptional activators are Sp1, Ets, AP1 and PPARy, and some repressor factors are TWIST, YB1 or Id1. Additionally, some of these factors serve as guides for epigenetic modifiers [96].

#### Oncogene-induced senescence

Cellular senescence can be initiated when mitogenic signals are excessive. This supposes an antitumoral mechanism that is activated by the overexpression of some protooncogenes and the loss of function of tumor suppressor genes. The first evidence of OIS was found after ectopic overexpression of an oncogenic version of HRAS (HRASG12v) in normal nonimmortalized cells. These cells stopped proliferating and acquired a phenotype similar to cellular senescence with high expression of p16^INK4a^ and ARF/p53 [97]. *RAS* is a member of the protein kinase MAPK signaling pathway, which can activate OIS involving p53 and p16^INK4a^ in the response [98]. Furthermore, hyperactive mutated versions of other proteins of this signaling pathway, such as RAF, MEK and BRAF, and many protooncogenes, such as *PI3K*, *AKT*, *MYC*, *ERBB2*, and *p38 MAPK*, can induce a mitogenic signal that activates OIS. Similarly, loss of function of tumor suppressor genes, such as *PTEN* or *NFI*, initiates senescence [4, 20].

The expression of oncogenes and hyperactivation of mitogenic signals induce DNA damage and start the DDR. In cells overexpressing mutated HRAS, a hyperreplicative phase that ends in the cell cycle stops induced in OIS with partially replicated DNA is observed [99]. This replicative stress induced by oncogenic activation contributes to the genomic instability of tumoral cells. The mechanisms proposed for this are diverse (replication factors or nucleotides decrease, increase or decrease origin firing, resulting in a reduction in the ratio of elongation forks) [100–102]. Recently, a study identified and compared the replication origin sites generated before and after the activation of the oncogenes *CCNE1* and *MYC*. Replication origins were located inside of highly expressed genes after overexpression of both oncogenes. Replication forks collapsed in these cases due to the conflict generated between transcription and replication, and it was also associated with DBs and chromosomal rearrangement breakpoints in the cellular model mentioned and in patient samples [102].

Evidence suggesting that OIS is a physiological phenomenon is abundant in the scientific literature and it has been obtained from animal models and tumor samples from patients. For example, acquired and congenic melanocytic nevi (benign lesions derived from melanocytes) show acid β-galactosidase activity, high expression of *p16*^*INK4a*^ and absence of MIB1 without proliferative activity [103, 104]. Furthermore, in transgenic mice that overexpress only *BRafV600E*, proliferative arrest was observed in melanocytes, while if the tumor suppressor *Pten* was silenced in these mice, melanomas were generated [105, 106]. Similarly, in the lung, a model of inducible expression of oncogene *KRas-V12* was used to study neoplastic lesions. Acid β-galactosidase activity, *p16*^*INKa*^ expression and SAHFs were observed in adenomas. However, in the few adenocarcinomas generated, these senescence markers were absent [107]. Similarly, a *Pten* knockout model showed senescence markers in prostate early-stage neoplasia but not in malignant tumors generated after silencing *Trp53* [108]. When *NRAS* was overexpressed in lymphoid cells of mice, OIS evidence was found. Lymphomas were produced when an additional p53 mutation or Suc39h1 (histone methyltransferase enzyme that mediates *pRb* silencing) mutation was induced along with the oncogenic overexpression of *NRAS* [109]. Additionally, in the pituitary gland of mice, senescence markers have been found after *E2F* overexpression, with proliferation and tumorigenesis stopping [110]. Furthermore, microadenomas have been found in this gland in humans with a prevalence of 10-5% with evidence that OIS could stop the development of these lesions [111].

#### Therapy-induced senescence

Senescence-induced therapy (TIS) is initiated in tumoral cells and other cells of patients as a consequence of radiotherapy, chemotherapy, or targeted therapy against cancer disease [112, 113]. Evidence of TIS has accumulated since the discovery of OIS. Several studies have shown the relevance of p53 and p16^INK4a^ in the induction of senescence in tumors after treatment and its relationship with prognosis *in vivo* [114–116]. This finding broke the paradigm of the immortality of tumoral cells and the belief that they do not senesce [117].

Most treatments against cancer disease produce DNA damage in tumoral and nontumoral cells of patients. This initiates DDR and, possibly, senescence. It has been described that ionizing radiation can induce TIS in a dose- and cell type-dependent manner. At the molecular level, the cell cycle stop induced by ionizing radiation depends on *p21*^*CIP1*^ and the mutational state of p53, as has been shown in breast cancer, colorectal cancer, and glioblastoma [118, 119]. Furthermore, ionizing radiation can increase ROS production in cells, especially mitochondrial ROS, damaging DNA. It is also known that AKT protein can contribute to ROS increase and senescence initiation via p53 after radiotherapy treatment [120]. *In vivo,* ionizing radiation damages hematopoietic progenitors in bone marrow due to an increase in ROS [121].

Inducing senescence in tumors has positive effects, including proliferative stop and apoptosis in tumoral cells and, therefore, tumor suppression. It has also been suggested that paracrine induction of senescence could be beneficial for tumor treatment [113]. This has been verified in tumoral cells, but their response to SASP-conditioned medium differs among cell lines [122]. However, TIS can induce protumoral effects in the microenvironment. For example, SASP production, specifically IL6 and IL8, can induce a malignant and invasive phenotype in epithelial premalignant cells. Additionally, tumoral cells can exit the senescence phenotype, resulting in relapse in patients. These cells are called dormant cells and have been detected, among others, in prostate, breast, colon, lung cancer and glioblastoma [123–128].

#### Oxidative stress-induced senescence

There are many different theories that connect oxidative stress, cellular senescence and aging. Most of them have been developed since Dr. Denham Harman proposed the free radical theory of aging in 1956. According to this theory, “aging must be related to the attack of free radicals, which are generally products of metabolic processes, to cellular components” [129]. Later, this theory was focused on the production of free radicals in the mitochondria, and it was proposed that free radicals’ deleterious effects on the cell were caused by the prooxidant-antioxidant imbalance, in favor of oxidation [130].

The most common free radicals produced in the normal metabolism of the cell are superoxide anion (O_2_^-^) and hydroxyl radical (^-^OH). Specifically, dysfunctional mitochondria are characterized by electron leakage, which generates O_2_^-^ as a secondary byproduct, especially in the reactions produced in complex I (NADH dehydrogenase) and complex III (cytochrome bc1 complex). Different kinds of molecules, such as lipids, proteins and DNA, are damaged by these radicals. When oxidative stress reaches high levels over long periods of time, cells acquire a senescence-like phenotype. Furthermore, ROS accumulation seems to contribute to OIS induction, TIS and p16^INK4a^-mediated senescence [131].

DNA can be damaged by ROS in multiple ways: single-base damage, double string crosslinking, DNA-protein adducts, and double string bounds [132]. Additionally, ROS have been related to telomere shortening [133]. Thus, ROS can initiate DDR and, consequently, cell senescence. It has also been proposed that the existence of a positive feedback loop between DDR and ROS is promoted by the long-term activation of *p21*^*CIP1*^ by p53. Moreover, p53 can activate other prooxidant genes, such as *TP53I3* (*PIG3*); as a consequence, mitochondria are damaged, and electron leakage increases [134, 135].

Proteins are another target of ROS contributing to cellular oxidative stress. ROS play an important role in cellular signaling through oxidation-reduction of amino acids, generally methionine and cysteine residues. However, some oxidative modifications are not enzymatically reversible, such as carbonylation. When these irreversible modifications accumulate in the proteins of the cells and there is an excess of oxidated proteins, cells try to degrade them via proteosomes. Nevertheless, oxidated proteins bound by crosslinking inhibit proteosomes and accumulate in lysosomes [131, 136].

There are several mechanisms in the cell to avoid damage produced by ROS. This regulation of homeostasis is performed by endogenous antioxidant molecules such as glutathione and enzymes such as superoxide dismutases, catalases and thiol peroxidases. Additionally, cells can use exogenous antioxidant molecules such as micronutrients and vitamins [131]. Some studies indicate that a lack of antioxidant enzymes contributes to premature aging. For example, *PrxIII*-deficient mice show reduced physiological capacities compared to wild-type mice [137]. Reduced expression of *PrxI* accelerates the appearance of hemolytic anemia and tumors in mice [138]. In human fibroblasts, the expression of sulfoxide reductases is reduced during replicative senescence [139]. Moreover, nonenzymatic antioxidant molecules seem to contribute to senescence delay. The balance of oxidated/reduced glutathione grows in favor of oxidation with age in blood and in stem cells from adipose tissue in humans [131, 140]. However, noncytotoxic high doses of synthetic antioxidant compounds induce premature senescence in mesenchymal stem cells [141].

The role of p53 in responding to oxidative stress depends on ROS levels. On the one hand, p53 promotes the generation of ROS when oxidative stress is persistent. On the other hand, under transient or mild oxidative stress, p53 can activate the transcription of genes with antioxidant functions, such as GPX1, SOD2, TIGAR, SESN1, SESN2 and SLC2A9 [135].

Despite all of the evidence and classic theories, the number of studies that question the deleterious effect of ROS in senescence and aging has recently grown. It has been observed in yeast and *C. elegans* that an increase in ROS and oxidative stress do not necessarily accelerate aging [142]. In mice, genetic modification to increase mitochondrial ROS production does not accelerate aging, and the increase in antioxidants does not extend the lifespan of the animals [143].

Several compounds and enzymes with antioxidant activity can extend the lifespan of human fibroblasts by reducing ROS and mitochondrial damage and decreasing telomere shortening. Nevertheless, the addition of some of these compounds to the culture medium can increase ROS production. This is explained by the ability of antioxidants to be oxidized. Something similar is reported in studies that show how culture of many types of cells in hypoxia can increase lifespan but also increase mitochondrial ROS production [144]. However, the role of ROS in cellular signaling and homeostasis has gained attention, as it can be implicated in proliferation and cell survival in certain circumstances. For this reason, it has been proposed that even though ROS participate in the signaling of damage caused by aging, perhaps its accumulation with age is not a cause [145].

**Fig. 1:**
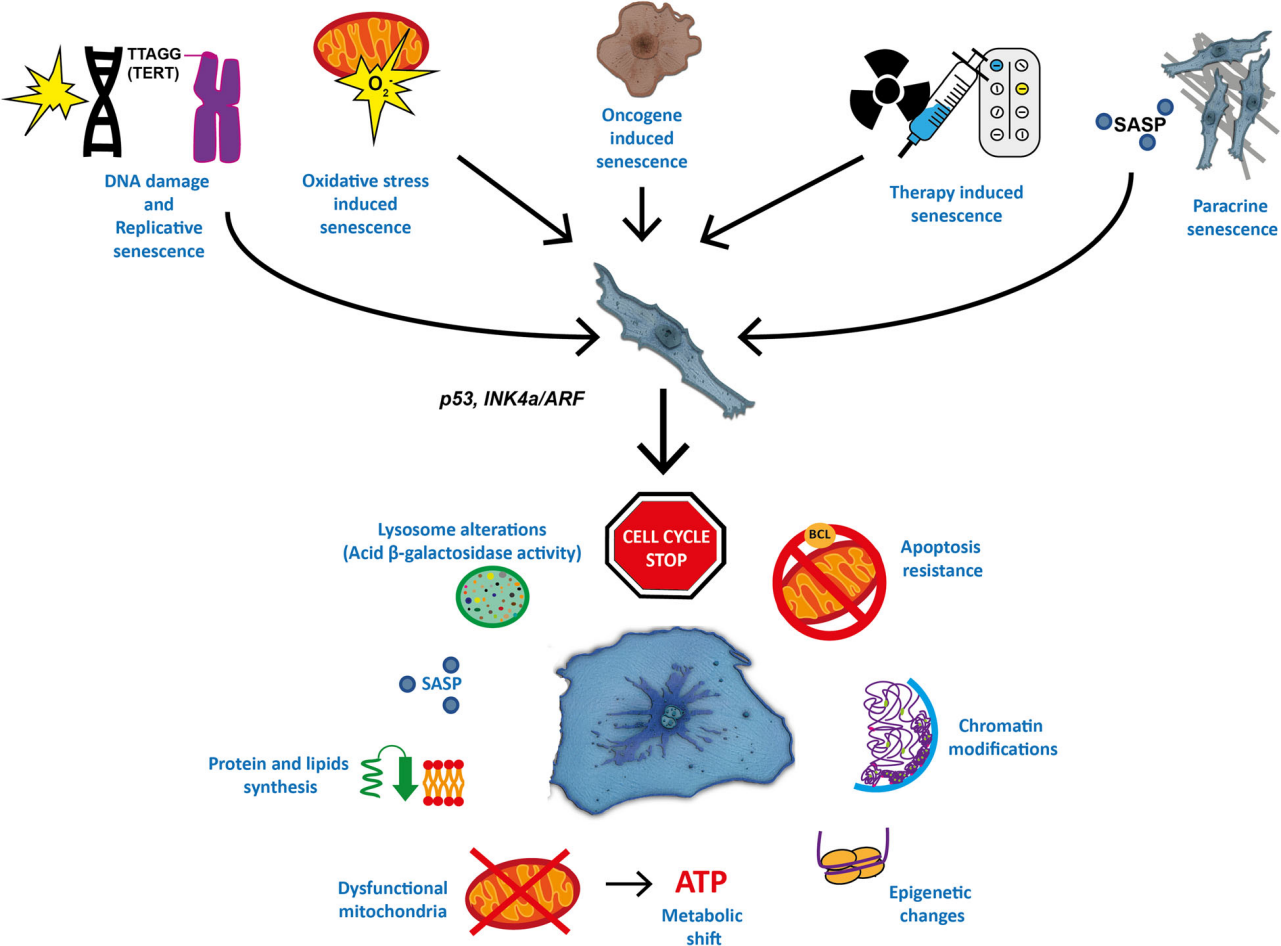
General mechanisms of cellular senescence. Cellular senesce can be induced by several stimuli like DNA damage, telomere shortening, oxidative stress, gain of oncogenes and loss of function of tumor suppressor genes, therapy against cancer disease and, additionally, SASP paracrine signaling. Response to these stimuli is mediated by the main effectors of cellular senescence which are p53 and genes translated from *INK4a/ARF* locus. Consequently, senescent cells develop a complex phenotype, which most representative feature is cell cycle stop. Additionally, other features of cellular senescence are represented

To understand the dual role of ROS in cells, the mitohormesis theory has been developed as a stress and signaling mediator. This theory suggests that different kinds of cellular stress can initiate mitochondria-related signaling by which cells increase their tolerance to ROS, unfold proteins and other deleterious elements for cell homeostasis. In this response, the activation of antioxidant mechanisms is important [146] (Fig. 1).

### Hypoxia, cellular senescence and immortalization

#### Mechanisms for physiological adaptation to hypoxia

**Fig. 2:**
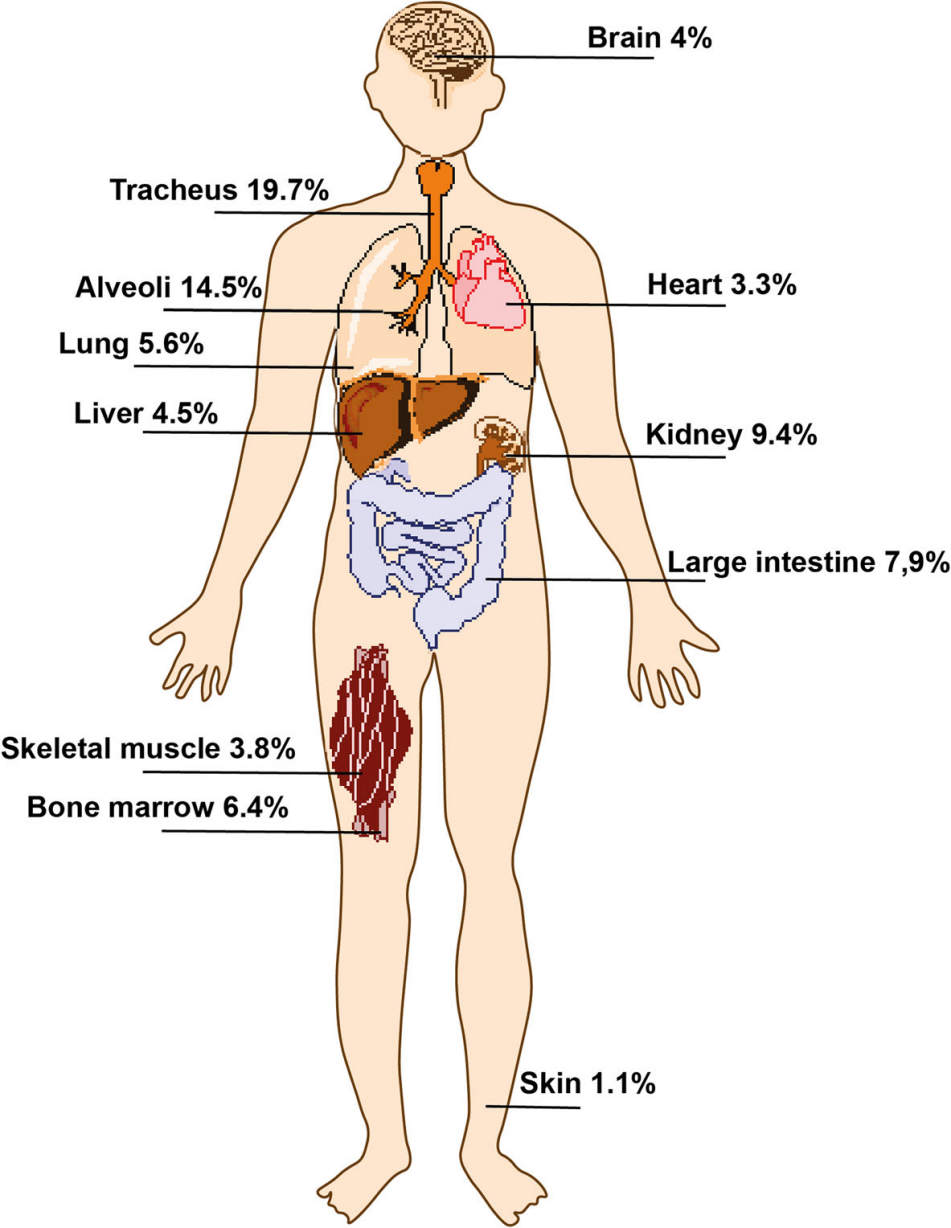
Oxygen levels in different organs of human body. Oxygen levels diminish after caption from atmosphere by respiratory organs and distribution along circulatory system to different organs. Oxygen concentrations in human organs are expressed in percentages here

What is the effect of environmental oxygen? Atmospheric oxygen levels are approximately 20%; however, availability in our organism is not the same. Oxygen should be distributed from the respiratory system to every tissue through blood flow, and consequently, not every cell is exposed to the same oxygen concentration. For example, the oxygen concentration in the human brain is near 4%, similar to skeletal muscles or the liver. The heart presents a value of 3.3%, and the lungs, despite being the organs responsible for oxygen intake, are supplied with oxygen levels of 5.6%. Other organs, such as the smooth intestine or kidneys, show levels higher than 8%, and in the skin, by contrast, oxygen only reaches 1% [147, 148] (Fig. 2).

There is both a systemic and a molecular response to hypoxia in mammals. The complete physiological response consists of an acute reflex of hyperventilation and sympathetic activation to incorporate more oxygen and distribute throughout the whole organism [149]. To develop this, there are specialized organs containing oxygen-sensitive cells, such as carotid bodies [150, 151]. Any circumstance or pathology that could mean an interruption in the caption and distribution of oxygen can trigger this response. For example, oxygen concentration decreases with geographic altitude, which triggers a systemic adaptive response [152].

Additionally, at the pathological level, failures in breathing, oxygen flux or hemoglobin levels can trigger a response to hypoxia. Some of the diseases that can activate the physiological mechanisms mentioned are chronic obstructive pulmonary disease (COPD), sleep apnea, lung fibrosis, ischemic cardiopathy and chronic cardiac failure. Moreover, it has been demonstrated that other conditions and pathologies, such as hypertension, diabetes, and some hepatic and renal diseases, are also related to hypoxia [149, 153].

As mentioned, there are adaptive mechanisms to hypoxia at the molecular level. The most relevant is the mechanism carried out by hypoxia inducible factors (HIFs). This molecular pathway can be activated in every cell and depends on the stabilization of HIFα subunits (HIF1α, HIF2α or HIF3α) in hypoxia [154]. When this happens, HIFα subunits dimerize with HIF1β, also known as ARNT, and are translocated to the nucleus. Once there, the heterodimer binds hypoxia response elements (HREs) (G/ACGTG) in the DNA and promotes the expression of the target genes [155, 156].

The most important regulation of HIFα protein is posttranslational, and it is performed mainly by prolyl-hydroxylase enzymes (PHDs), which are sensitive to oxygen levels. There are 4 isoforms of these enzymes, among which PHD2 is the most important, and its optimal activation range is under normoxia and moderate hypoxia. PHDs hydrolyze 1 or 2 proline residues that are well conserved among HIFα proteins, making these proteins susceptible to ubiquitination mediated by von Hippel-Lindau protein (VHL). This last posttranslational modification marks HIFα for degradation in proteosomes. Moreover, the factor inhibiting HIF1α (FIH), another oxygen-sensitive protein, also hydrolyzes HIFα residues. In this case, however, modifications are made in asparagine residues and at lower oxygen levels than PHDs. This inhibition is mostly specific to HIF1α because HIF2α is, in comparison, resistant [156, 157].

**Fig. 3:**
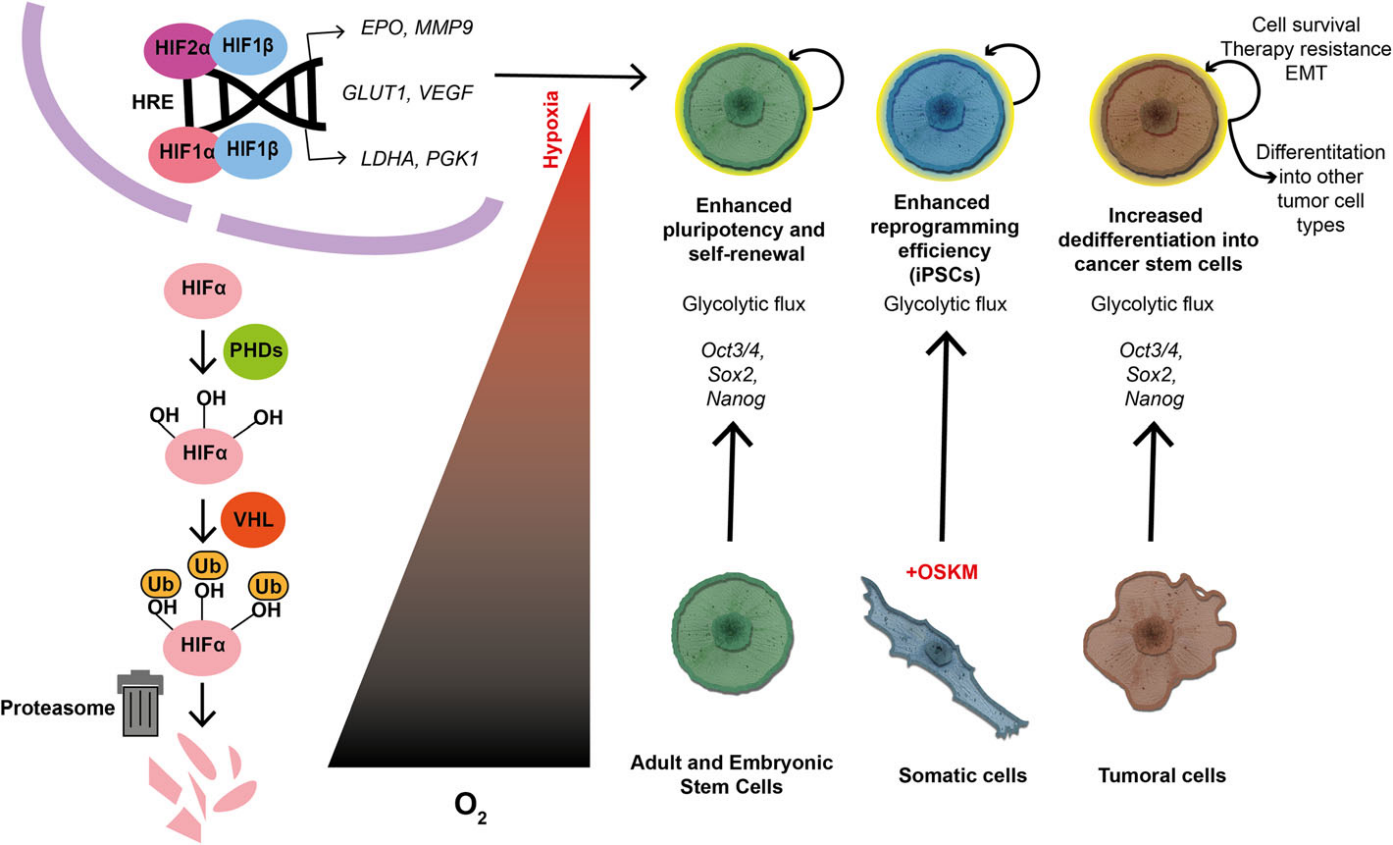
HIF signaling and effects of hypoxia on stem cells. In normoxia, HIFα proteins can be hydroxilate by PHDs, this allows ubiquitination mediated by VHL and its degradation in proteasome. However, when oxygen levels are low, HIFα proteins are stable, and they can translocate to nucleus where they dimerize with HIF1β and bind to HRE sequences in the DNA. HIF dimers activate transcription of hypoxia response genes. Through this mechanism and others, hypoxia promotes glycolytic flux and expression of stemness-related genes supporting stem cell properties in adult and embryonic stem cells, iPSCs and cancer stem cells. OSKM: Yamanaka factors *Oct3/4*, *Sox2*, *Klf4* and *cMyc*

There are notable functional differences between HIF1α and HIF2α isoforms. While HIF1a is considered a ubiquitous protein, the activity of HIF2α is more specific to different cell types, such as cardiomyocytes, endothelial cells, hepatocytes or glial cells. Furthermore, in many cases, HIF1α has been identified as responsible for the acute response, while HIF2α has been described as responsible for the chronic response and geographic altitude response. Both proteins share some common transcriptional targets, such as *GLUT1* or *VEGF*, but they specifically regulate others, such as *LDHA* and *PGK1*, in the case of HIF1α and *EPO* and *MMP9* and *OCT4* in the case of HIF2α. These specificities depend on the cellular context and oxygen concentration [158] (Fig. 3). Additionally, there is a third HIFα subunit, called HIF3α, whose expression in adult tissues is located in the thymus, brain, lungs, heart and kidneys. This isoform presents alternative splicing variants, and in some of them, there is no transactivator domain, as there is in HIF1α and HIF2α. For this reason, HIF3α is thought to be an inhibitor of HIF-dependent transcription. In particular, one of the splicing variants, called IPAS, can bind HIF1α and form a heterodimer that is unable to activate the transcription of HRE-containing genes [157, 159].

#### Relationship between molecular response to hypoxia and cellular senescence

Hypoxia is a feature of locally advanced tumors, and a lack of vascularization deprives cells from oxygen. Furthermore, hypoxia promotes tumor progression and is a marker for poor prognosis in patients [7, 9]. However, it is not yet clear whether hypoxia works as a stressor inducing cellular senescence or can facilitate immortalization prior to malignant transformation, contributing to tumorigenesis. In this respect, several studies describe how different oxygen concentrations, especially low levels of oxygen, can alter the lifespan of cells. In the case of human fibroblasts, low oxygen levels extend lifespan by 20%, while bovine fibroblasts increase lifespan by 80% and 500% in fibroblasts derived from mice [160–163] (Table 1).

**Table 1:** Lifespan increase in different cellular models cultured under different oxygen concentrations

Cellular model	O_**2**_ concentration	Lifespan increase	Reference
IMR90, WI38	2 and 10%	25%	Packer et al. Nature, 1977
IMR90	1, 6 and 12%	22%	Saito et al. Exp. Cell Research, 1995
Mouse embryonal fibroblasts (MEFs)	3%	500%	Parrinello et al. Nature Cell Biology, 2003
Bovine embryonal fibroblasts (BFF)	2%	80%	Betts et al. Biogerontology, 2008

The increase in the lifespan of MEFs cultured under hypoxia compared to normoxia has been justified by the difference in accumulated oxidative damage. MEFs proliferated in hypoxia despite conserving the wild-type p19^ARF/^p53 pathway, which is essential for avoiding immortalization [164], and expressing high levels of *p16*^*INK4a*^. Moreover, MEFs from mice deficient in different proteins involved in DNA repair stopped proliferating with elevated signs of DNA damage when they were cultured in 3% O_2_. However, they continued proliferating when telomerase activity was suppressed. Therefore, it has been suggested that DNA damage accumulation induces senescence in MEFs under normoxia. When the p19^ARF^/p53 pathway is altered in these conditions, cells become insensitive to this damage and are immortalized. Additionally, the existence of a mutagenic or adaptive response acquired in hypoxia has been suggested, which allows the extension of lifespan despite p19^ARF^/p53 and p16^INK4a^ activity. This adaptation only appears at a minimum frequency in cells under normoxia compared to hypoxia.

In contrast, it has been suggested that human cells initiate replicative senescence due to telomere shortening [165], and they have a greater capacity to prevent or repair DNA damage. This may explain why the lifespan increase produced by hypoxia in human cells is lower than that produced by MEFs. This hypothesis tries to explain the different susceptibility to cancer and aging speed between the two species [162]. Nevertheless, this extreme sensitivity to oxygen damage seems to be a particular feature of mouse lab strains, perhaps related to very long telomeres. Therefore, there might be alternative mechanisms altering lifespan in mammals, as oxygen sensitivity does not correlate with lifespan in other species [166]. It has also been proposed that an increase in glycolysis contributes to reducing oxidative damage and can facilitate cellular immortalization. An increase in the expression of *PGM* can bypass replicative senescence and *Ras-*induced senescence in MEFs due to the increase in glycolytic flux [167]. Alternatively, this lifespan extension has been causally associated with different cellular signaling pathways, especially the HIFα pathway. HIF1α and HIF2α directly target genes that can regulate the cell cycle, such as *p21*^*CIP1*^ and *MYC* [168, 169]. It has been suggested that HIF1α can activate the expression of *hTERT* in the presence of mitochondrial ROS [170]. Moreover, p53 inhibition can occur in a HIFα-dependent and HIFα-independent manner [171], and suppression of the conversion from proliferative arrest to irreversible senescence (a processed named *geroconversion*) can be independent of HIFα and p53 [172].

Among the mechanisms proposed to promote senescence via HIF1α, the cell cycle stops through activation of *p21*^*CIP1*^ and *p27*^*KIP1*^ [173–175] or inhibition of *CDC25A* [176]. Additionally, hypoxia is related to SASP and, consequently, to paracrine and autocrine induction of senescence. HIF1α can activate the transcription of multiple genes that are relevant for SASP, such as *IL8*, *CXCR2*, *GRO*α, *IL6* and *PAI1*. Hypoxia has also been associated with senescence independent of HIF proteins. In anoxia, different pathways converge on the cell cycle stop without HIF interaction [177]. In cells with loss of *VHL*, senescence is induced by p400 and p27^KIP1^ [178]. Additionally, observation of senescent cells in mouse melanoma is related to high expression of *BCL2* in hypoxia [179].

### Hypoxia and stem cell capacity

#### Hypoxia and embryonic and adult stem cells

Hypoxia has also been associated with stem cells and pluripotency. The first evidence found was related to organismal development because embryos are exposed to a partial state of hypoxia during gestation [180]. The mammalian oviductal and uterine lumen are hypoxic (1.5% O2). This means that the preimplemented embryo is adapted to a low oxygen atmosphere. In humans, it has been shown that low oxygen atmospheres can produce high-quality embryos and improve implementation from developing zygotes [181]. Additionally, placental development is associated with hypoxia, as cytotrophoblasts, which are stem cells forming placenta, proliferate better at low oxygen concentrations *in vitro* [182, 183]. Moreover, it has been demonstrated that embryonic and somatic stem cells reside in a hypoxic niche. This niche generates a microenvironment in which oxygen levels are even lower than those in tissues where they are located (1-8% O2) [184].

ESCs can be maintained *in vitro* indefinitely when they are cultured under specific conditions. These specific conditions try to activate the main signaling pathways of pluripotency and self-renewal. In ESCs, the activity of the transcription factors Oct3/4, Sox2 and Nanog, which form the core of the pluripotency circuit, is essential. Oct3/4 can bind DNA to control the expression of multiple genes related to pluripotency. Oct3/4 can form a heterodimer with Sox2 and regulate the expression of genes such as *Lefty1*, *Fgf4*, *Oct3/4*, *Sox2* and *Nanog*. Furthermore, Oct3/4 binds to enhancers of pluripotency genes, and its expression in ESCs is tightly regulated because very high or low levels of this protein can induce differentiation. Nanog can work in coordination with Oct3/4 and Sox2, but its expression levels fluctuate in ESC populations. Among its transcriptional targets, it is possible to find genes related to pluripotency, such as *Esrrb1*, *Rif1*, *Foxd3* and *Rest*. In addition to these 3 transcription factors, other proteins play an important role in the maintenance of ESC capacities, such as SMAD1 or STAT3, and some coordinators of its activity, such as Klf4, Esrrb, cMyc and Tfcp2 l1. Generally, the action of all of these proteins leads to the activation of signaling pathways such as LIF/Stat3, Wnt/β-Catenin, FGF/ERK, TGF/SMAD and PKC [185].

Multiple studies have demonstrated that hypoxia promotes self-renewal and pluripotency maintenance in ESCs and other types of stem cell cultures. It is known that self-renewal, pluripotency and the expression of stem cell markers and effectors such as *Nanog, Oct3/4* and *Sox2* are promoted by hypoxia, either dependent or independent of HIFα. For example, it has been shown that low oxygen concentrations promote neural crest and hemopoietic stem cell survival and prevent ESC differentiation. *In vitro,* hESCs benefit from low oxygen conditions, reducing differentiation and increasing the expression of pluripotency genes. MYC, OCT4, SOX2 and NANOG are upregulated by HIF2a in these conditions, promoting pluripotency. Additionally, it has been shown that HIF2a can increase glycolytic flux under 5% oxygen, upregulating CTBP1 and CTBP2 to promote self-renewal [181].

Despite all evidence generated, the influence of hypoxia on the differentiation and dedifferentiation capacity of these cells is still controversial. This is probably because of the variability generated by multiple factors, such as the maturation state of the stem cells, hypoxia exposure time or oxygen levels used [8, 186–188]. Hypoxia culture can promote the differentiation of mESCs toward endothelial cells, simulating the physiological process of embryonic vascular differentiation [181]. Indeed, the pluripotency markers *Oct3/4*, *Tra1-60*, *Nanog* and *Sox2* are downregulated under high levels of ROS. This promotes ESC differentiation into mesodermal and endodermal lineages through MAPK signaling pathways. This effect of ROS in ESCs can be counteracted by the addition of antioxidant compounds to the cell culture [189].

### Hypoxia and induced pluripotent stem cells

Mammalian cells can be reprogrammed to induce pluripotent stem cells (iPSCs) through transduction of transcription factors *Oct3/4*, *Sox2*, *Klf4* and *cMyc* (OSKM), also known as Yamanaka factors [190, 191]. However, the application of this biological resource in cell therapy is currently limited. This is due to low efficiency in the iPSC generation process and the oncogenic risk entailed in reprogramming protocols. This risk involves the activation of protooncogene *cMyc* and the use of virus, which can alter the genome when exogenous genetic constructs are randomly inserted in receptor cells [192, 193]. It has been demonstrated that hypoxia increases reprogramming efficiency and reduces the number of transcription factors needed to achieve reprogramming, but it is still unknown how this effect is produced [10]. Several studies have proposed that different molecular effectors are responsible, but discussion has been focused on HIFα proteins. Among the mechanisms described, the metabolic shift from mitochondrial oxidation to glycolysis, which is common to stem cells and iPSCs, is relevant. For this metabolic reprogramming, it seems that HIF1α and HIF2α are both necessary and that their activation increases reprogramming efficiency and pluripotency. Nevertheless, the activation of each HIFα factor occurs differently in every reprogramming stage, and stabilization of HIF2α in the final reprogramming stages inhibits iPSC generation [8, 194–196].

First, cellular senescence acts as a barrier for cellular reprogramming. This was determined due to the activation of the main effector pathways of cellular senescence (p53, p16^INK4a^/pRB, p21^CIP1^, DDR and *INK4a* locus remodeling) after overexpression of Yamanaka factors. Another important piece of evidence is the silencing of the *INK4a/ARF* locus in mature iPSCs and ESCs. It has been demonstrated that inhibition of these cellular senescence effectors can increase reprogramming efficiency in human and mouse fibroblasts, although it entails oncogenic risk [197, 198].

Later, it was observed that senescence could promote reprogramming efficiency *in vivo* via paracrine action of SASP. When Yamanaka factors are overexpressed, the senescence barrier is activated in tissues, and only some cells allow reprogramming. Senescent cells initiate the secretion of SASP factors, including IL6. The secretion of this interleukin and the activation of its downstream targets increase the reprogramming efficiency of nonsenescent surrounding cells. In this signaling process, activation of the *INK4a* locus, which induces a senescent phenotype with expression of *IL6, is necessary*. However, it has been demonstrated that p53, *Arf* and p21^CIP1^ are not necessary in this process. This increase in reprogramming efficiency induced by senescence is observed in senescence induced by damage to tissues and in aging [199–201].

Recently, more evidence about this relationship between senescence and reprogramming has been uncovered. It has been demonstrated that cyclic and continuous overexpression of Yamanaka factors interferes in epigenetic remodeling associated with aging in mouse models [202]. Chemotherapy-induced senescence mediated by p53 and the epigenetic marker H2K9me3 contributes to the acquisition of stem cell properties in tumoral cells *in vivo* [203]. Furthermore, it has been observed that SASP promotes cell plasticity and tissue regeneration. When senescent cells were inoculated into the livers of mice, liver stem cell markers were increased [204].

### Hypoxia and cancer stem cells

Tumors are heterogeneous entities composed of cells with different identities, including cancer stem cells (CSCs). CSCs are characterized by self-renewal and differentiation in other types of tumoral cells. They can initiate a tumor individually. Although CSC and stem cell capacities are similar, the mechanisms regulating these capacities are deregulated in CSCs [205]. CD34^+^/CD38^-^ cells were first identified in 1997 as unique cells able to regenerate myeloid leukemia in mice [206]. Since then, CSCs have been isolated from breast, colon, brain, and many other tumors [7].

CSCs are not a static identity in tumoral heterogeneity, as they are considered a cellular state. Cells in the tumor are differentiated and dedifferentiated to this cellular state, and the tumor microenvironment plays an essential role. For example, it has been demonstrated that CSCs can influence nearby fibroblasts and transform them into cancer-associated fibroblasts (CAFs). These CAFs can promote the WNT and NOTCH pathways, contributing to CSC identity maintenance [207, 208]. Stromal mesenchymal stem cells promote CSC maintenance through activation of the NFκB signaling pathway and secretion of CXCL12, IL6 and IL8. Additionally, CSCs can modulate the microenvironment secreting TGFβ to mimic the stem cell niche microenvironment, promote epithelial-mesenchymal transition (EMT) and modify distal niches secreting VGFA, TGFβ, TNFα and LOX.

Hypoxia is part of the tumoral microenvironment, and it is relevant in locally advanced tumors in which vascularization is unable to provide proper flow of oxygen and nutrients for every cell. This lack of oxygen initiates a survival response. The hypoxia response contributes to tumor progression, angiogenesis, metabolic reprogramming, modulation of immune response, metastasis, and therapy resistance. For these reasons, hypoxia clinically correlates with tumor aggressiveness and poor disease prognosis. Additionally, mediators of the hypoxia response at the molecular level have been considered therapeutic targets [7, 9].

As mentioned, different signaling pathways participate in the response to this microenvironment condition, but HIFα signaling is the most studied. It is also known that tumors that conserve normal oxygen conditions have some oncogenes, such as *AKT*, and the loss of some tumor suppressor genes, such as *PTEN*, *PML*, and *TSC,* can also activate translation of HIFα proteins [7]. It has been demonstrated that hypoxia can promote dedifferentiation and CSC property maintenance. Among these genes promoted by hypoxia, *OCT3/4*, *SOX2*, *NANOG* and *KLF4* are important, but the NOTCH pathway and epigenetic modifiers such as BMI1 and SIRT1 also contribute to this effect [9, 209–211]. Furthermore, it seems that HIF1α can also counteract the cMYC effect, stopping the cell cycle and maintaining CSC identity [173].

There is a direct correlation between dormant CSCs and hypoxia. In the hypoxic and necrotic regions of tumors, cells show a dedifferentiated phenotype and dormant state, while they are present in vascularized regions [125]. Thus, it has been proposed that hypoxia can activate a dormant state to increase cell survival in CSCs exposed to stress conditions. For example, in glioblastoma cells, hypoxia activates *PP2A,* which mediates the dormant state program, inducing cell cycle arrest in G1/S phase [128]. In the prostate, HIF1α can activate the expression of *CXCR4* and *NDRG1*, which is regulated by nMYC to induce a dormant state [126, 127]. Furthermore, *HIGD1A*, a transcriptional target of HIF1a, can promote a dormant state through the production of ROS [124]. This dormant state shares some features with cellular senescence like the cell cycle stop and, also a secretome similar to SASP, which includes factors like IL1-a, IL1-b, IL-6, IL-8, CXCL1 and CXCL2. This secretome may contribute to cancer progression [212]. For instance, it has been shown that prostate cancer cells can induce a transient senescence phenotype with proliferative arrest through SPARC secretion. This senescent stay can be considered dormant state and correlates with bad prognosis [213]. There is evidence that CSC secretes different SASPs programs [212, 213]. This could mean that CSCs, which do not experience senescence and are resistant to chemotherapy, could produce a different secretome and escape destruction by the immune system, that remove instead senescent cells from the tissues. A hypoxic microenvironment could, in the same way, influence the secretome, therefore altering the behavior of the immune system in the site.

Furthermore, in physiologically hypoxic environments, senescent cells express lower levels of pro-inflammatory SASP factors [214]. This is due to AMPK activation induced by hypoxia, which in turn lead to AMPK-dependent suppression of the mTOR-NFκB signaling, thus regulating SAPS secretion [214]. Since the mTOR maintained high activity seems important for “geroconversion”, its decreased activity induced by low oxygen could be important in the bypass of senescence by hypoxia. These results could indicate that one pathway, rather than pleiotropic signaling, can control senescence physiology through SAPS. These results underline the important role of SAPS in malignancy and resistance to therapy.

Hypoxia signaling and ROS have been related because both can activate CSC stress signaling through the TGFβ and TNF pathways to promote cell survival and maintenance of CSC identity and, additionally, promote EMT. Furthermore, TGFβ can stabilize HIFα proteins. Moreover, HIF1α can increase the synthesis of glutathione in response to chemotherapy stress, which promotes the acquisition of the CSC phenotype [7].

It has been proposed that hypoxia can alter the CSC population in tumors in two different ways: promoting dedifferentiation of tumor cells and limiting the differentiation of CSCs. In breast primary carcinoma, hypoxia increases the population of CD44^+^/CD24^-^ and increases ALDH^+^ through the HIF1α and AKT/β-catenin signaling pathways [215]. However, in ER-negative breast cancer, it seems that PHD3 activates the NFκB pathway, increasing the CD44^+^/CD24^-^ population in hypoxia [216].

Both isoforms, HIF1α and HIF2α, have important functions in hypoxia and stem cell property acquisition in tumors. Nevertheless, it seems that tumors preferentially express the HIF2α isoform. In glioma cell lines, there is a subpopulation of cells with greater migration capacity. These cells express high levels of *SOX2* and *OCT3/4*, which are induced by HIF2α [217]. Additionally, in gliomas, the cell membrane marker CD44 cleaves and releases its cytoplasmic fraction. This fragment of protein binds HIF2α to increase the expression of stemness-associated genes [218]. In a mouse model of *MYC*-induced leukemia, NANOG and SOX2 facilitate MYC binding to the HIF2α promoter, which maintains the CSC state through inhibition of p53 and ROS production [219]. Furthermore, HIF2α can activate the expression of *LIF,* and the expression of both genes is positively correlated in patients with colorectal cancer [220].

It has been demonstrated that EMT provides high aggressiveness to tumoral growth because cells acquire CSC properties, motility, invasive capacity and dissemination, senescence resistance, therapy resistance and resistance to the immune system [221]. Hypoxia favors metastasis, increasing the expression of genes related to EMT, whose expression is frequent in CSCs. Hypoxia can activate the transcription of *SNAI1*, *ZEB1*, *TWIST*1 and *TCF3* through HIF1α [222, 223]. In breast cancer, hypoxia promotes the expression of *ZEB1,* and it inhibits MYB to promote EMT. Furthermore, the expression of EMT genes is necessary for the appearance of circulating tumor cells (CTCs). These cells can cause metastasis and present CSC properties. CTCs with CSC features have been found in the bloodstream of patients with different types of tumors. For example, hepatocarcinoma CTCs are CD45^-^/ICAM1^+^, CTCs in prostate cancer are CD133^+^ and those in breast cancer are ALDH^+^. In many cases, CTCs found in patients express *OCT3/4*, *SOX2* and *NANOG* [7]. Moreover, hypoxia can modulate the secretion of vesicles to increase the expression of GTPase associated with endosomes, *RAB22A*, and the exosome marker *CD63*. These exosomes in hypoxia usually contain molecules such as VEGFR2, TNFα1, β-catenin, AKT and EGFR and contribute to tumor progression, angiogenesis, immune suppression, invasion, and metastasis [224–226].

Tumors show high glycolytic activity in substitution for oxidative phosphorylation even under normal oxygen conditions and despite its lower efficiency. This phenomenon is known as the “Warburg effect” and involves the widespread consumption of glucose as a consequence [227]. This offers an advantage under hypoxia, which is reinforced by the adaptive response mediated by HIF inducing the expression of key glycolytic enzymes and inhibiting mitochondrial metabolism. HIF1α can promote the activation of PDK1, inhibiting the conversion of pyruvate into acetyl-CoA and inhibiting flux to the tricarboxylic acid cycle (TCA). HIF1α can activate the transcription of *LDHA,* which converts pyruvate into lactate and limits flux to acetyl-CoA. Furthermore, HIF1α promotes the transcription of *PKM2*, which is the last step in glycolysis, and it also works as a coactivator of HIF1α to promote glycolysis and tumor growth [9]. Transformed cells express alternative isoforms of key glycolytic enzymes compared to normal cells because of HIF1α activation. For example, HIF1α induces the expression of *HK2* and *ENO1* and helps to inhibit apoptosis and promote cell migration. Moreover, HIF1α can induce the expression of the *GLUT* glucose transporter [228, 229]. These transporters have been found to be overexpressed in CSCs in glioblastoma, ovarian cancer and pancreas, and they can promote self-renewal and tumor initiation capacities [230].

Hypoxia has also been related to epigenetic modifications that promote tumor progression. For example, it has been shown that hypoxia can modulate TET activity, which induces *de novo* methylation in the sequence of genes associated with EMT, such as *INSIG1* [168, 231]. Hypoxia can modulate the action of histone modifiers such as HDAC3 and WAD5, which mediate EMT in head and neck cancer and in breast tumors [232]. Additionally, miRNAs such as *miR-21*, *miR-200* and *miR-210* are expressed in hypoxia and regulate EMT [233–236]. It is also known that HIFα proteins interfere with the regulation of the expression of *ZNF127* and *ALKBH5,* which can demethylate the 3’-UTR of *KLF4* and *NANOG* mRNA, increasing their expression in breast tumors [237] (Fig 3).

## Conclusions

Oxygen levels are heterogeneous in mammalian tissues, and external and internal conditions can produce additional variations. For this reason, mammals have developed physiological and molecular responses to low levels of oxygen. Current knowledge about cellular senescence has been experimentally developed at atmospheric oxygen concentrations. This may be a bias because the cell lifespan can be extended under hypoxic conditions. Although oxygen damage sensitivity has been proposed to be responsible for different lifespan extensions between humans and mice, it is still controversial. There might be alternative mechanisms controlling lifespan extension induced by hypoxia. Similarly, it is still under discussion whether hypoxia promotes differentiation or helps to maintain stem cell identity under certain conditions. Nevertheless, it has been widely demonstrated that culture of ESCs and some adult stem cells under hypoxic conditions helps to maintain self-renewal and pluripotency. Additionally, hypoxia contributes to the CSC pool and correlates with cancer malignancy and poor prognosis. Therefore, we speculate that evidence generally supports the role of hypoxia as a proliferation and dedifferentiation stimulus whose mechanisms should be studied in depth to understand cellular senescence, stem cell biology and cancer disease.

## Data Availability

No datasets were generated during the current study.

## Abbreviations

CAFs: Cancer associated fibroblasts
CDKs: Cyclin dependent kinases
CKIs: CDK inhibitors
COPD: Chronic obstructive pulmonary disease
CSCs: Cancer stem cells
CTCs: Circulating tumor cells
DDR: DNA damage response
DNA-SCARS: DNA segments with chromatin alterations reinforcing senescence
DSBs: Double string breaks
EMT: Epithelial-mesenchymal transition
ESCs: Embryonic stem cells
FIH: Factor inhibiting HIF1α
HIFs: Hypoxia inducible factors
HREs: Hypoxia response elements
iPSCs: Induced pluripotent stem cells
OIS: Oncogene induced senescence
OSKM: YAMANA factors *Oct3/4, Sox2, Klf4* and *cMyc*
PcG: Polycomb group of proteins
PHDs: Prolyl-hydroxylases
pRB: Retinoblastoma protein
PRC2: Polycomb repressive complex 2
ROS: Reactive oxygen species
SADSs: Satellite distensions associated to senescence
SAHFs: Senescence associated heterochromatin foci
SASP: Senescence associated secretory phenotype
TASCC: TOR autophagy spatial coupling compartment
TCA: Tricarboxylic acid cycle
TIS: Therapy induced senescence
VHL: Von Hippel-Lindau protein
